# Enhancing sustainable agri-food systems using multi-nutrient fertilizers in Kenyan smallholder farming systems

**DOI:** 10.1016/j.heliyon.2023.e15320

**Published:** 2023-04-11

**Authors:** Ivan S. Adolwa, James Mutegi, Joses Muthamia, Angela Gitonga, Samuel Njoroge, Abednego Kiwia, Dismas Manoti, Franklin S. Mairura, Eileen B. Nchanji

**Affiliations:** a African Plant Nutrition Institute, C/o IFDC, ICIPE Compound, Duduville, Kasarani, P.O Box 30772-00100, Nairobi, Kenya; b Alliance for a Green Revolution in Africa (AGRA), West End Towers, Waiyaki Way, P.O. Box 66773 Westlands, Nairobi 00800, Kenya; c Tegemeo Institute, Tetezi Towers, George Padmore Road, P.O. Box 20498, Nairobi, Kenya; d University of Embu, Department of Water and Agricultural Resource Management, 6-60100 Embu, Kenya; e International Center for Tropical Agriculture

**Keywords:** Multi-nutrient fertilizer blends, Micro-nutrients, Profitability, Sustainable crop production, Food security, Yield response

## Abstract

Persistent food insecurity in the global south has triggered calls for sustainable development worldwide. Moreover, more than a quarter of the world's population suffers from micronutrient deficiencies or hidden hunger. The population bulge, declining soil fertility and inadequate/inappropriate use of farm inputs in Sub-Saharan Africa place it in a precarious position. Multi-nutrient fertilizer blends have been mooted as a key innovation in closing yield gaps and boosting food and nutrition security. This study assessed the extent of multi-nutrient fertilizer blends utilization and yield response across agroecological zones and their on-farm profitability under Kenyan smallholder farmer conditions. We collected data through a detailed household survey conducted in eight counties in Kenya representative of high, medium, and low productivity zones using a sample of 1094 smallholder farmers. Multi-nutrient fertilizers increased maize yields significantly (*P < 0.05*), eliciting a 400% yield increase compared to the control and 108% greater maize yield than conventional fertilizers in the high potential zone. Conversely, at 3.7 t/ha conventional fertilizers elicited a significant (*P < 0.05*) yield response in Irish potatoes in the high potential areas. Multi-nutrient fertilizers increased on-farm profitability of crops, specifically for potato production systems where a benefit: cost ratio (BCR) of more than 2 was observed. Farmers may break even when they use multi-nutrient fertilizers on maize particularly in the low potential areas. Therefore, there is considerable potential for multi-nutrient fertilizers to increase crop productivity while being economically viable across agroecological zones and cropping systems. However, the uptake of multi-nutrient fertilizers among farmers is quite low across the country, except for small pockets where limited interventions have been carried out. This calls for sustained efforts to scale multi-nutrient fertilizers with a focus on clear messaging that stresses the need to apply appropriate rates of various nutrients including the secondary nutrients and micro-nutrients.

## 1 Introduction

In Sub-Saharan Africa (SSA), low soil fertility, land degradation, and inadequate/in appropriate use of farm inputs has resulted in sub-optimal crop production [1,2]. In addition, the effects of increased temperature, erratic rainfall, frequent floods, and prolonged droughts due to climate variability and change has negatively impacted on agricultural productivity in the SSA region [3]. Consequently, crop production in the majority of SSA, including Kenya, is characterized by large yield gaps between attainable and actual farm yields [[4], [5], [6], [7], [8]]. Closing these yield gaps is key if food security is to be attained in SSA. Projections indicate that for Africa to feed herself by 2050, the yield level (for cereals) must increase to about 7 t ha^−1^ or an average of 136 kg ha^−1^ year^−1^ [9]. Therefore, a key challenge for researchers and development practitioners in Africa is to design systems that can innovatively and sustainably raise farm-level crop productivity.

Crop productivity enhancement interventions targeting the use of multi-nutrient fertilizer blends and other innovations aimed at improving access to favorable input and output markets can tackle the twin challenge of lack of food and hidden hunger [10]. The recent increases in fertilizer prices and other factors including biophysical variability have an important impact on the profitability and efficiency of fertilizer use [11,12], which influences farm level decisions on fertilizer investments. Interventions to increase crop productivity in smallholder farms of the SSA region should therefore recognize local variability in agro-ecological conditions and crop response to fertilizers [13].

In this study, multi-nutrient fertilizer blends (henceforth multi-nutrient fertilizers) are defined as those that contain the macronutrients such as Nitrogen (N), Phosphorus (P) and Potassium(K) as well as secondary nutrients like Sulphur (S), Magnesium (Mg), and/or Calcium (Ca) and micronutrients such as Zinc (Zn), Copper (Cu), Selenium (Se), Manganese (Mg), Boron (B) and Molybdenum (Mo). The application of multi-nutrient fertilizers has the potential of improving crop yields by addressing multiple soil nutrient deficiencies [14,15]. Moreover, micronutrient applications to crops are linked to their improved concentration in the consumable crop products, and the subsequent improved health of consumers of such products [16,17]. Biofortification of crops such as sweet potatoes, cassava, maize, beans, and pearl millet with micronutrients has been used for managing health problems associated with micro-nutrient deficiencies e.g., stunted mental and physical growth, anemia, impaired immunity, and night blindness, particularly in children and women in resource-poor rural areas [[18], [19], [20]].

However, for such interventions to be sustainable it is crucial to understand the economic and livelihood impacts of multi-nutrient fertilizers on smallholder farmer households [[21], [22], [23]]. While studies on the economic impacts of conventional (straight and compound) fertilizers as well as hybrid seeds in SSA farming systems abound [[24], [25], [26], [27], [28], [29], [30]], there is scant information on on-farm economic impacts of crop-specific multi-nutrient fertilizers, particularly in the Kenyan context. In Kenya, fertilizer supply shocks were experienced in 2020 due to multiple factors including the COVID-19 related supply chain disruptions, high input prices (e.g., natural gas), reduced fertilizer production in Europe, and export restrictions from China [31]. This resulted in 50–60% (2020–2021) fertilizer price increases in Kenya, following global increases in fertilizer prices [32]. Maize production in Kenya (2020–2021) declined by an estimated 550,000 metric tons (MT) because of fertilizer price increases (resulting in lower application rates) and failing rainfall (the most severe drought in 40 years [31]). Therefore, there is need for an investigation of the economic benefits derived from use of multi-nutrient fertilizers at the farm level especially against a backdrop of recent shocks experienced in the fertilizer supply chain.

A wide range of multi-nutrient fertilizers, which come in different brands depending on the company of manufacture, are currently available in the Kenyan market. Table 1 highlights the most common multi-nutrient fertilizer products available in the Kenyan market as of 2020. However, the extent of farmer awareness and use of these fertilizers in comparison to conventional fertilizers is not widely known. In addition, the available fertilizer used by small-scale farmers in Kenya is usually not the correct type needed for various crops and soils, and most farmers are not aware of their soil quality, correct application rates, timing for application, and placement in the soil-plant continuum [2,33].

**Table 1:** Some multi-nutrient fertilizers for cereals, legumes, and potatoes available in the open Kenyan market in the year 2020 [6,34].

Fertilizer Company and Formulation	Brand Name/Use	Main Target Crop/s
*Yara compound fertilizers*		
NPK 23-10-5 +2 MgO +3 S + 0.3 Zn	YaraMila Cereal-Planting	Cereals e.g. maize and sorghum
NPK 13-24-12+4 S + 0.01Zn	YaraMila Power -Planting	Cereals, potatoes, vegetables
NPK 17-29-6 +6 S + 0.2Zn	Yara MiCROP Planting	Cereal crops
NPK 40-0-0 + 5.5 S	YaraVera Amidas -Topdress	Cereals e.g. maize, rice, barley
NPK 24-0-0 +6 S	YaraBela Sulfan-Topdress	All crops
NPK 40-0-0 +5 S + 0.6Zn	Yara MiCROP Topdress	Cereal crops
NPK 15-9-20 + 1.8 MgO +9.5 SO_3_ +0.015 B + 0.02 Mn + 0.02 Zn	YaraMila Winner-Topdress	Fruits, vegetables, potatoes
*OCP-Kenya Ltd*		
NPSB 18-38-0 +6 S + 0.01 B	Planting	Cereals
*MEA Fertilizers Ltd. Nakuru, Kenya*		
NPK 10-26-10 +2CaO +5MgO +3 S	Planting	Cereals
NPK 14-26-6 +4CaO +5 S	Planting or Topdress	Fruits and vegetables
NPK 10-22-20 +9 S + 0.7MgO	Planting	Rice
NPK 26-0-0 +13CaO	Topdress	Mainly cereals and vegetables
*Toyota Tsusho Fertilizers Africa, Eldoret*		
NPK 14-29-6 +S + CaO + MgO + Zn + B	Baraka Planting Standard	Cereals e.g. Maize
NPK 32–0 -3 +9 S +3CaO +1MgO	Baraka Topdress Standard	Cereals e.g. Maize
NPK 14-28-14 +S + CaO + MgO + Zn + B	Baraka Planting for Potatoes	Potato, onions, tomatoes
NPK 18:0:21: +S + CaO	Baraka Topdress for Potato	Potato, onion, tomatoes
NPK10:25:14+3 S+5CaO+1MgO+0.2Zn+0.1 B	Baraka Planting for Legume	Legumes e.g. beans, green grams
*ARM Ltd. Athi River*		
NPK 10-26-10 +Ca, Mg, S, Zn, Cu, Mn, B, Mo	Mavuno Planting	Maize, Sugarcane, Wheat
NPK 26-0- 0 +Ca, S	Mavuno Topdress	General Topdress
*Fanisi Fertilizer Ltd*		
NPK 13-26-10+ Ca + Mg + +S + Zn + Cu + B + Mn	Fanisi Mazao Planting	Maize
NPK 14-27-13 + Ca + Mg + S + Zn + Cu + Bo + Mn	Fanisi Mazao Planting	Potato
NPK 20-10-20 + Ca + Mg + S + Zn + Cu + Bo + Mn	Fanisi Mazao Planting	Rice
NPK + Ca + S	Fanisi Mazao Topdress	Vegetables/horticultural crops
NPK 14-10-18 + Ca + Mg + S + Zn + Cu + Bo + Mn	Fanisi Mazao Planting (Hortimax)	Vegetables/horticultural crops
*Export Trading Group, Mombasa*		
NPK 18:38:0 + S + Ca + Mg + Zn + B	KynoNafaka (Planting)	Cereal crops
NPK 15-29-0 + Ca, Mg, S, K, Zn, B	Kyno Planting	Cereal crops
NPK 15:9:21 + Ca, Mg, S, K, Zn, B	Kyno Horti	Horticultural crops
Agrotain urea-AS blend 40:0:0 + 6 S	KynoPlus “S” Topdress	Primarily cereals

The main objective of this study is to furnish evidence about the use, crop yield responses and the profitability of using multi-nutrient fertilizers at specific rates (kg/ha) under smallholder farmer conditions in Kenya's low, mid, and high potential agro-ecological zones (AEZs). The distribution of counties along varying climatic gradient and cultures across the Kenyan landscape is key to covering varying input demand levels and performance. Key questions revolve around crop yield responses -for Kenyan smallholder farming systems and the profitability of blended multi-nutrient fertilizers on smallholder Kenyan farms.

## 2 Materials and methods

**Fig. 1:**
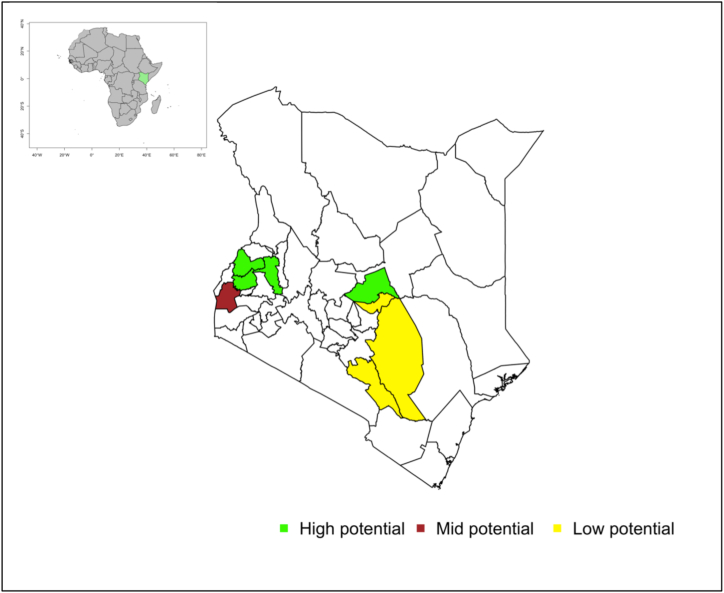
Map of the study counties (Inset: The map of Africa with Kenya highlighted in green). Note: Shapefiles sourced from the database of country administrative areas (GADM).

Data was collected through a detailed household survey conducted in eight counties between the months of December 2020 and February 2021. These counties located in the republic of Kenya include: Makueni, Kitui, Tharaka-Nithi, Siaya, Meru, Uasin Gishu, Kakamega and Bungoma. The eight counties are representative of the low, medium (or mid) and high potential agro-ecozones of Kenya (Fig. 1).

### 2.1 An overview of the study counties

#### 2.1.1 The high potential zone

The high potential zone includes the counties of Bungoma, Kakamega, Uasin Gishu, and Meru. These counties are generally characterized by high and reliable rainfall, and relatively fertile soils, and are subsequently considered as the breadbasket (or potential breadbasket) counties of Kenya. A brief characterization of each of these counties is presented below.

Bungoma County in western Kenya is home to 1,670,570 people and has a population density of 552 persons per km^2^ (https://kenya.opendataforafrica.org). Agriculture is its economic backbone, with the county being the fourth largest producer of maize (*Zea mays)* and common beans (*Phaseolus vulgaris*) in Kenya. Bungoma is characterized by relatively fertile soils and receives adequate and well distributed bi-modal rainfall, suitable for small scale agriculture. About 70% of its 183,000 ha is considered arable [35].

Kakamega County, also in western Kenya, has a population of 1,867,579 and a population density of 618 persons per km^2^ (https://kenya.opendataforafrica.org). Agriculture in Kakamega county is mainly characterized by mixed farming of maize and legumes such as common beans, although cash crop farming is prevalent in the humid upper midland (Tea; *Camellia sinensis*) and sub-humid midland (Sugarcane; *Saccharum officinarum*) agroecological zones. The county has a bi-modal rainfall pattern and receives up to 2000 mm of rainfall per annum with area bordering the Kakamega forest, the last remnant of equatorial forest in Kenya, receiving the highest amounts [36].

Uasin Gishu County is situated in the mid-west of Kenya's rift valley. It has a total population of 1,163,186 persons and a population density of 343 persons per km^2^ (https://kenya.opendataforafrica.org). The county is a highland plateau with a generally undulating landscape. It has a total land area of 334,500 ha, 90% of which is arable and receives rainfall in the range of 900–1400 mm per annum [36]. Uasin Gishu is a top producer of the staple crop maize as well as wheat and milk. The county is usually referred to as the breadbasket of Kenya because it has relatively high and reliable rainfall, comparatively large farm holdings and highly mechanized farming [37].

Meru County is situated on the eastern and northern slopes of Mt. Kenya (Kenya's highest mountain). It has a total population of 1,545,714 persons and a population density of 221 persons per km^2^ (https://kenya.opendataforafrica.org). Meru is an agricultural county, which has favorable weather conditions for both cash crop and subsistence farming. Main food crops include maize, common beans, Irish potatoes (*Solanum tuberosum*), wheat (*Tricum* sp.)*,* cabbages (*Barassica oleracea* var. *capitata*), tomatoes (*Solanum lycopersium*) and carrots (*Daucus carota* subsp. *Sativus*)*.* The county receives a significant amount of rainfall up to 2800 mm per annum, which is bimodally distributed [38].

#### 2.1.2 The mid potential zone

The mid-potential zone was represented by Siaya County located in western Kenya. The county has a smaller population and population density than the previous two western Kenya counties (i.e., Kakamega and Bungoma), standing at 993,183 persons and 393 persons per km^2^. Siaya is an agricultural county with the main activities revolving around fishing, farming, and livestock production. The farming system here is pre-dominated by subsistence crop-livestock systems with maize as the dominant crop. The county receives an average of about 1800 mm of rainfall per annum that is bi-modally distributed. However, uneven rainfall patterns, droughts and degraded low to moderately fertile soils constrain agricultural productivity and food security in the county [39].

#### 2.1.3 The low potential zone

The low potential zone was represented by Tharaka Nithi, Kitui, and Makueni counties. A brief characterization of each of these counties is presented below.

Tharaka Nithi County neighbors Meru County but is much smaller in terms of population at 393,177 persons and has a population density of 153 persons per km^2^. It is also drier, with the semi-arid zone receiving only about 700 mm per annum of rainfall [40]. The upper zone has a higher productivity level of maize, beans, tea, and coffee, whereas in the lower zone green grams, millet, sorghum, and black beans can be grown productively.

Kitui County is also in the low potential agricultural zone with the drier ecological zones receiving only 450 mm of rainfall per annum and the subhumid zone about 1000 mm [41]. It is a large county geographically (30,429 km^2^), with a population of 1,136,187 persons. Thus, its population density of 37 persons per km^2^ is much smaller than for the other counties.

Makueni county is to the east of Kitui County and has a population of 987,653 and a low population density of 121 persons per km^2^. Makueni is relatively wetter, with the lower midland zone receiving as much as 1200 mm of rainfall per annum [42]. Smallholder mixed farming systems are prevalent in Makueni and Kitui and crops such as maize, beans, green grams (*Vigna radiata*), pigeon peas (*Cajanus cajan*), sorghum (*Sorghum bicolor*) and millet (*Panicum miliaceum*) are commonly grown [43].

### 2.2 Sampling strategy

We employed a multi-stage sampling strategy entailing several stages. First, we purposively selected eight out of the fourteen counties where soil fertility and plant nutrition management interventions were carried out by the African Plant Nutrition Institute (APNI). These interventions entailed establishment of on-farm fertilizer demonstrations and training of farmers, extension officers and other agricultural stakeholders on the use of multi-nutrient fertilizers. The counties selected were representative of the main AEZs across the country. Within the selected eight counties, we purposively selected sub-Counties and wards where soil fertility and plant nutrition management interventions had been implemented. Subsequently, we randomly selected respondents from 2 villages per ward using the skip interval method [44]. To arrive at a balanced sample, we sampled farmers from a village reached with interventions on multi-nutrient fertilizers and improved seeds and a non-participant one not reached with these interventions. Within each farm, data was collected on fertilizer use at plot level.

The sample size for the study was 1094 respondents whereby 120 farmers were selected in Kakamega, 120 in Bungoma, 121 farmers in Uasin Gishu, and 151 farmers in Meru for the high potential zone, 121 in Siaya for the mid potential zone, and lastly 149 farmers in Tharaka Nithi, 161 farmers in Makueni and 151 in Kitui for the low potential agro-ecozone. Face to face interviews between respondents and trained enumerators were conducted using a structured questionnaire after consent was given by the respondents (Table 2). In total, there were 512 farmers sampled in the high potential zone, 121 farmers in the mid-potential zone and 461 farmers in the low potential agro-ecological zone. The questionnaire was deployed using the mobile phone-based application, SurveyCTO, for fast, efficient, and accurate data collection. The tool contained several sections to capture data on household socioeconomics and demographics, farm characteristics, crop production, livestock husbandry, social capital, and food and nutrition security. Therefore, the data was collected at plot, household, and farm levels. For instance, crop production data (inputs used and costs, yield) was disaggregated at plot level. This was done because of within-farm variability in soil fertility and crop management. At household level we collected typical demographic data including age, gender of household head, household size, household monthly income, years of schoolingand household assets. At the farm level, data on livestock production and utilization of livestock products was collected. Questions were directed to the respondents so as to measure key dimensions of food security such as food availability and access [45,46]. Therefore, the respondents were queried on whether they were able to get enough food, ate preferable foods, ate smaller portions, skipped meals, or had no food to eat at all. Also, the frequency on how often they experienced these incidences was captured. A reference period of 12 months was used for all economic activities.

**Table 2:** Sample size distribution of farmers sampled in different Counties and AEZ in Kenya.

County	AEZ			Across County
	High-potential	Mid-potential	Low-potential	
Bungoma	120	–	–	120
Kakamega	120	–	–	120
Kitui	–	–	151	151
Makueni	–	–	161	161
Meru	151	–	–	151
Siaya	–	121	–	121
Tharaka-Nithi	–	–	149	149
Uasin Gishu	121	–	–	121
**Across AEZ**	**512**	**121**	**461**	**1094**

### 2.3 Fertilizer nitrogen rate

Inorganic fertilizer inputs for maize, bean, and potato crop enterprises (input quantity [kg] and land application areas [ha]) were recorded in all fields at plot level putting into consideration the fertilizer blend used for each crop and the field sizes for first and second fertilizer applications. The applications were consequently converted into N application rates (kg N ha^−1^) using nutrient concentrations for each of the fertilizer inputs that were enumerated from farmers (Table 3). The calculation was implemented in an Excel worksheet with the fertilizer data using a *VLOOKUP* procedure that integrated the fertilizers and their N rates as a link table (Table 3). The nitrogen input contributions from first and second fertilizer applications were summed and divided by field application areas to derive the nitrogen application rate for each field during the cropping season. The *VLOOKUP* procedure was also used to create the fertilizer N rate factor (Nitrogen rate levels), which classified the total nitrogen applications into 3 categories (0–30 kg Nha^−1^, 30–60 kg Nha^−1^ and >60 kg Nha^−1^). Fertilizers were also classified into 3 categories of fertilizer types using the *VLOOKUP* procedure including the control, conventional and multi-nutrient fertilizers. The control referred to fields that farmers did not apply any fertilizer inputs. Multi-nutrient fertilizers include mineral fertilizers which contain two or more of the macronutrients N, P and K and small amounts of secondary nutrients (S, Mg or Ca) and micro-nutrients such as B or Zn. In addition, conventional fertilizers include mineral fertilizers with the primary macronutrients but without secondary nutrients and micro-nutrients (see Table 1, Table 3).

**Table 3:** Fertilizers applied by farmers, their Nitrogen contents (%) and fertilizer types.

Fertilizer	% N	Fertilizer type
NPK (23:23:0)	23	Conventional
NPK (17:17:17)	17	Conventional
Baraka standard Planting	14	Multi-nutrient
Baraka standard topdess	25	Multi-nutrient
Baraka legume	10	Multi-nutrient
Baraka Standard topdress	32	Multi-nutrient
Baraka Potato planting	14	Multi-nutrient
Baraka Potato Topdress	18	Multi-nutrient
Baraka Legume planting	10	Multi-nutrient
Baraka Horticulture	7	Multi-nutrient
Fanisi Planting	13	Multi-nutrient
Fanisi Topdressing	15	Multi-nutrient
Kyno Nafaka	18	Multi-nutrient
KynoPlus S	46	Multi-nutrient
Kynoplus Top	46	Multi-nutrient
DAP	18	Conventional
CAN	27	Conventional
MEA NPK 10:26:10	10	Conventional
NPS (OCP)	18	Multi-nutrient
NPSB (OCP)	18	Multi-nutrient
UREA (46:0:0)	46	Conventional
Folia Feeds	24	Multi-nutrient
Mavuno Planting	10	Multi-nutrient
Mavuno-Top Dress	26	Multi-nutrient
Mijingu Nafaka	9	Multi-nutrient
YaraMila Winner	15	Multi-nutrient
YaraMila Power	13	Multi-nutrient
Yara Mila Cereals	23	Multi-nutrient
YaraVera Amidas	40	Multi-nutrient
YaraBela Sulfan	26	Multi-nutrient
YaraMila Cereal	23	Multi-nutrient
YaraBela Extran	33.5	Multi-nutrient

On average, the field sizes for maize plots were 0.9 ha, potato plots 0.6 ha and beans plots 0.7 ha. Conventional fertilizers were used on 53% of maize fields, multi-nutrient fertilizers were used in 6% of maize fields, and 41% of maize fields were unfertilized (Table 4). Potatoes were more commonly fertilized with conventional (73%) and multi-nutrient fertilizers (18%). Bean crops were rarely fertilized (86% of bean fields), while conventional fertilizers were more commonly applied on beans (12%), compared to multi-nutrient fertilizers (2%). Maize fields recorded higher N application rates from multi-nutrient fertilizers in both the first and second applications across AEZs (Table 4).

**Table 4:** First and second fertilizer N applications for various crops across AEZs.

Crop	Fertilizer type	Field size (ha)	First fertilizer (kg N/ha)	Second fertilizer (kg N/ha)
*Maize*	Control	1.2 (674)	0.0	0.0
	Conventional	0.7 (883)	22.9	22.3
	Multi-nutrient	0.7 (94)	29.1	44.3
	Across maize	0.9 (1651)	–	–
Beans	Control	0.8 (695)	0.0	0.0
	Conventional	0.7 (95)	19.5	10.5
	Multi-nutrient	0.4 (18)	11.9	13.7
	Across beans	0.7 (808)	–	–
Irish potatoes	Control	0.5 (14)	0.0	0.0
	Conventional	0.5 (115)	49.6	27.1
	Multi-nutrient	0.9 (28)	41.7	35.9
	Across Irish potatoes	0.6 (157)	–	–
Number of fields	2616			

Values in parenthesis are number of fields (plots).

### 2.4 Data analysis

The R program (version 4.0.3) was used for statistical analysis, graphics, and generation of maps. The data was subjected to cleaning prior to analysis, during which the *EnvStats* R package was used to check for outliers using the *rosnerTest* procedure. To assess statistical differences in yield between fertilizer types and fertilizer application rates, the Levene's test for homogeneity of variance using R procedures was implemented using the *leveneTest* function (*car* package), with a threshold value of p = 0.05. Thus, variances were declared homogenous when the significance was p > 0.05 and subjected to the Tukey LSD test (*agricolae* R package, *LSD. Test* function). In addition, the Kruskall-Wallis post-hoc test was used when the assumptions of normality and homogeneity of variances were not met (p < 0.05), using the *kruskal* function*, agricolae* R package [] [47](Table 5). The Levene's test of homogeneity of variance showed that only maize (mid) and potato yield data (all high potential agro-ecological zone) met the assumptions of homogeneity and thus subjected to the Tukey LSD test, for fertilizer type and fertilizer rate comparisons. In addition, bean yields in the mid and high potential zone and potato yield met the homogeneity assumptions, thus subjected to the Tukey LSD test. The post hoc tests were significant for maize ANOVA (yield x fertilizer type) in the low and high potential zone (Kruskall-Wallis). For the fertilizer rate ANOVA model, maize (all zones, Kruskall-Wallis), and potato (Tukey LSD test) recorded significant results (Table 4). For fertilizer N, only the potato and bean (mid agro-ecological zone) data met the homogeneity assumptions.

**Table 5:** Tests of homogeneity of variance and post-hoc test for crop yields in different AEZs by fertilizer type and fertilizer rate.

Crop	AEZ	Levene's Test for Homogeneity of Variance *[step 1]*				Post-hoc tests *[step 2]*	
		Yield		Fertilizer N	BCR	Crop yield	
		df	F value, Pr (>F)			Kruskal- Wallis	Tukey
	Fertilizer type						
Maize	Low	2	3.2, 0.042*	3.2,0.040*	1.7,0.184	***	*na*
	Mid	2	1.6, 0.202	10.5,4.98e-05***	0.8,0.370	*na*	*ns*
	High	2	42.2, 2.2e-16***	260.2,2.2e-16***	1.0,0.329	***	*na*
Bean	Low	2	3.2, 0.042*	3.2,0.040*	2.2, 0.108	*ns*	*na*
	Mid	2	10.5,4.982e-05***	3.2,0.040 *	2.8, 0.074	*ns*	*na*
	High	2	42.2, 2.2e-16 ***	260.2, 2.2e-16 ***	2.8, 0.060	*ns*	*na*
Potato	High	2	0.8, 0.470	1.9, 0.162	4.8, 0.001**	*na*	*ns*
	***Fertilizer rate***						
Maize	Low	2	25.9, 1.5e-11 ***	22.1,5.43e-10 ***	2.2, 0.108	*	*na*
	Mid	2	4.7, 0.010 *	18.8, 3.64e-08 ***	2.8, 0.074	***	*na*
	High	2	37.8, 2.2e-16 ***	169.8, 2.2e-16 ***	2.8, 0.060	***	*na*
Bean	Low	2	8.9, 0.000***	61.6, 2.2e-16 ***	0.9, 0.340	***	*na*
	Mid	2	0.4,0.549	0.05, 0.824	1.0, 0.334	*na*	*ns*
	High	2	na (only 0–30)	na (only 0–30)	1.5, 0.233	*na*	*na*
Potato	High	2	1.8, 0.162	11.05, 3.3e-05 ***	4.8, 0.009**	*na*	***

To assess profitability of fertilizer interventions, we computed the benefits to costs ratios (BCR) for three crops (Maize, Common beans, and Irish potatoes). Various cost items including inputs, labor, and other variable costs (e.g., cost of sacks) were used in the analysis. The *ggplot 2* and *ggpubr* packages were used to plot 2-way means with standard errors. The R base procedures were used to fit and plot linear models between crop yield and fertilizer nitrogen rates. Regression models were also fitted for BCR and fertilizer nitrogen rates and faceted by agro-ecological zone and fertilizer category factors. A polynomial 2 regression model was fitted for potato BCR and nitrogen application rates, due to the non-linear nature of the data using R base regression procedures.

Often, the adoption of fertilizers or improved seed is treated as a binomial process where it is either adopted or not adopted. If the binary outcome is adoption, it is denoted as 1 and if it is non-adoption, it is denoted as 0. The models available for analyzing binary choice problems are the linear probability model (LPM), and the probit and logit models. However, with the LPM (uses ordinary least squares as predictors) the assumption is that error terms are normally distributed, which is not feasible given the limited values of a dichotomous, dependent variable [48]. Other problems include the high propensity for the predicted values to lie outside interval 0 to 1 and large prediction errors. Probit and logit models, which use the maximum likelihood estimation (MLE) to give unbiased and efficient estimates of the probability of the dependent variable assuming a dichotomous value are more suited to such analysis [48,49]. The two models have statistical similarities thus the decision to use probit or logit hinges on personal preferences and experiences [48]. In our case, a probit model estimated adoption of multi-nutrient fertilizers using variables drawn from key field, farm, socioeconomic, demographic, and institutional indicators. These variables were checked for multi-collinearity using the variance inflection factor (VIF) test (<10). The Stata application (Stata 13) package was used for this analysis.

### 2.5 Limitations of the study

The results of this study emanate from a cross-sectional survey as opposed to on-farm research (OFR) trials as detailed in Ref. [50], hence making it challenging to attribute causal effect of multi-nutrient fertilizer use to outcomes such as crop yields and profitability. However, the detailed coverage of the analysis and the rich socio-economic data provide a solid basis for an experiment-based study based on randomized complete block designs or other OFR trials.

## 3 Results

### 3.1 Descriptive indicators of the study counties

Only a very small share of fields cultivated by farmers across the counties can be considered moderate to highly fertile (Table 6). Overall, majority of farmers except for those in low potential areas, use fertilizers but only a small share applies multi-nutrient fertilizers on their fields. Bungoma and Kakamega Counties manifested the highest share of farmers using multi-nutrient fertilizers. This was not entirely surprising as extension activities aimed at driving higher usage of multi-nutrient fertilizers are heavily focused in these two counties. Moreover, the County Government of Kakamega has for the past few years implemented conducive policies for the uptake of these fertilizers. At 2.4%, the share for Uasin Gishu was lower than expected given that it is home to one such fertilizer blending company (Table 6). This result could be attributed to limited extension activities and less conducive policies compared to Kakamega.

**Table 6:** Descriptive indicators for the study counties.

	High potential					Mid potential	Low potential			
	Meru	Uasin Gishu	Kakamega	Bungoma	Average	Siaya	Makueni	Kitui	Tharaka-Nithi	Average
*Field level indicators*										
Share of fields considered highly fertile (%)	5.6 (23.0)	16.1 (36.9)	0.7 (8.5)	0.0 (0.0)	5.0 (21.9)	2.5 (15.7)	14.8 (35.5)	6.5 (24.6)	0.7 (8.3)	7.7 (26.7)
Share of fields applied with multi-nutrient fertilizers (%)	9.2 (28.9)	2.4 (15.2)	13.8 (34.6)	20.7 (40.6)	11.8 (32.3)	2.5 (15.7)	0.0 (0.0)	0.3 (5.7)	4.3 (20.4)	1.3 (11.5)
Share of fields applied with fertilizers (%)	82.8 (37.8)	95.3 (21.3)	89.5 (30.8)	94.4 (23.1)	89.6 (30.6)	84.9 (35.9)	43.6 (49.6)	33.6 (47.3)	62.3 (48.5)	45.1 (49.8)
*Household level indicators*										
Adult HH members (no.)	2.7 (1.25)	3.4 (1.6)	2.6 (1.2)	2.6 (1.1)	2.8 (1.3)	2.8 (1.6)	2.7 (1.3)	3.3 (1.5)	2.3 (1.0)	2.8 (1.3)
Share of HH heads that are female (%)	17.5 (38.1)	16.5 (37.2)	38.1 (48.7)	18.1 (38.6)	21.6 (41.2)	37.8 (48.6)	16.1 (40.4)	30.5 (46.1)	12.7 (33.3)	20.4 (41.5)
Age of HH head (years)	52.2 (12.5)	51.5 (12.1)	49.8 (13.5)	49.5 (12.7)	51.0 (12.7)	55.2 (13.8)	52.1 (15.0)	54.0 (17.3)	51.6 (14.1)	52.6 (15.6)
Years of schooling	9.3 (4.8)	11.3 (4.3)	9.3 (4.4)	10.7 (4.2)	10.0 (4.6)	9.2 (4.1)	10.6 (4.4)	9.4 (4.5)	10.3 (4.2)	10.1 (4.4)
Monthly expenditure (Kshs.)	10418.3 (8792.9)	33603.8 (34420.9)	13418.6 (16649.9)	9886.1 (7910.7)	15434.2 (20287.2)	15424.6 (14,574)	18838.9 (13848.8)	18205.4 (13524.7)	13740.4 (16172.6)	17194.3 (14556.5)
*Farm level indicators*										
Parcel size (acres)	1.4 (1.0)	2.8 (4.6)	1.2 (1.1)	1.1 (1.3)	1.5 (2.3)	1.3 (2.2)	3.0 (2.3)	3.6 (3.4)	1.2 (0.9)	2.8 (2.7)
TLU	2.0 (1.4)	5.0 (5.2)	2.1 (1.8)	2.3 (1.5)	2.7 (3.0)	3.6 (2.7)	4.6 (3.4)	3.5 (3.7)	1.5 (1.2)	3.4 (3.3)
*Social capital, credit, and infrastructural indicators*										
Share of HHs with electricity access (%)	54.2 (49.9)	60.2 (49.1)	39.6 (49.0)	44.7 (49.8)	49.4 (50.0)	22.4 (41.8)	20.9 (40.7)	17.9 (38.4)	38.4 (48.7)	24.5 (43.0)
Share of HHs that belong to associations (%)	32.2 (46.8)	40.3 (49.2)	29.5 (45.7)	42.9 (49.6)	35.6 (47.9)	39.1 (48.9)	30.3 (46.0)	22.8 (42.0)	31.1 (46.3)	27.8 (44.8)
Share of HHs that received agricultural training (%)	29.2 (45.5)	39.8 (49.1)	46.2 (49.9)	39.8 (49.1)	38.0 (48.6)	43.2 (49.6)	27.4 (44.7)	34.8 (47.7)	32.0 (46.7)	31.6 (46.5)
Share of HHs that obtained credit (%)	9.4 (0.3)	29.9 (45.9)	41.1 (49.3)	9.8 (29.8)	21.2 (40.9)	31.5 (46.5)	20.2 (40.2)	22.5 (41.8)	29.5 (45.6)	23.8 (42.6)
*Food security and nutrition indicators*										
Share of HHs that are food secure (%)	65.0 (47.8)	97.6 (15.2)	17.8 (38.3)	41.0 (49.3)	53.8 (49.9)	70.7 (45.6)	39.0 (48.8)	16.1 (36.7)	55.3 (49.8	34.6 (47.6)
Share of HHs that received nutrition information (%)	30.3 (46.0)	17.1 (37.7)	37.5 (48.5)	34.9 (47.8)	30.7 (46.1)	16.7 (37.3)	26.7 (44.3)	26.2 (44.0)	17.1 (37.7)	23.9 (42.7)

At the household level, household heads had a similar level of education across-board, and most were male-headed, although about a third of the households in Kakamega and Siaya were headed by females (Table 4). Only about 20–40% of the households could access credit, be members of associations (farming, labor, saving and loans etc.) or had received agricultural training in the last 12 months. As expected, the share of those households that were food secure was lowest in the low potential zones. Nevertheless, only slightly over a half of the households in the high potential zone were food secure. Uasin Gishu, which is characterized by large-scale farms and has the largest average parcel size in the high potential zone (Table 6), recorded exceptionally high shares of respondents that were food secure, quite the opposite of the situation in Kakamega. Coupled with the relatively precarious food security situation, only a few households reported receiving food nutrition information.

### 3.2 Crop yield response rates to multi-nutrient and conventional fertilizers under smallholder conditions

**Fig. 2:**
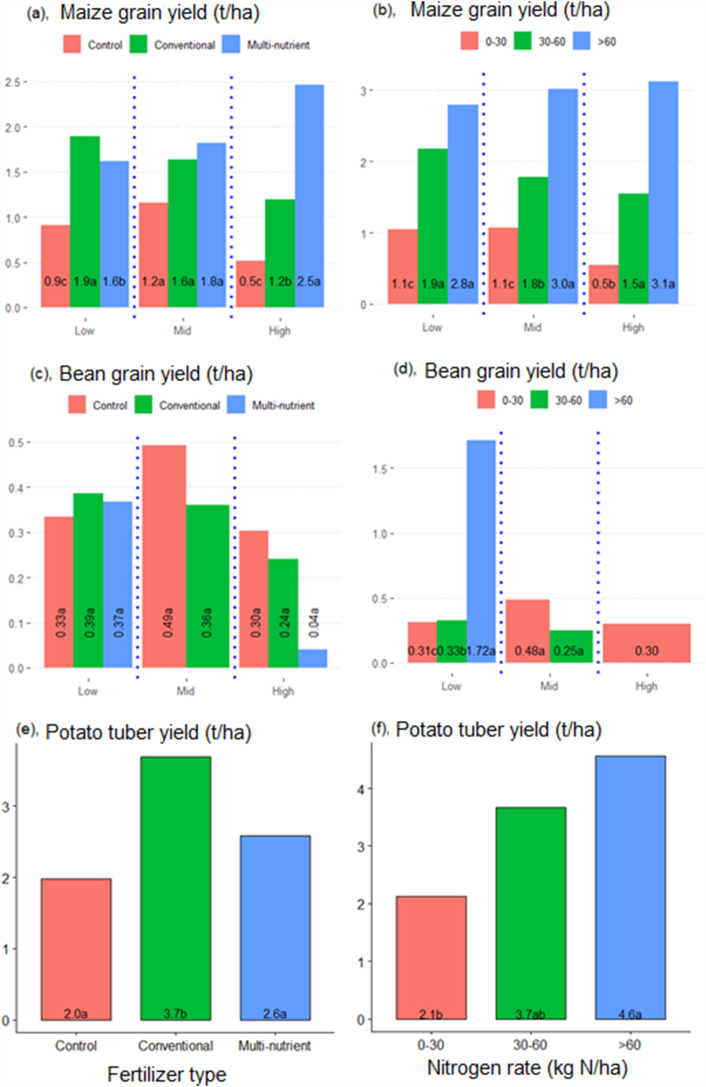
[a -f] Crop yield responses for different agroecological zones. Maize response by fertilizer types (A) and Nitrogen rate levels (kgN ha^−1^; B) for different AEZs. Bean response by fertilizer types (C) and Nitrogen rate levels (kgN ha^−1^; D) for different AEZs. Potato response by Nitrogen rate levels (kgN ha^−1^, F) and fertilizer types (E) for the high-potential AEZ.

Yield responses to multi-nutrient fertilizers were positive for the maize crop, particularly in high potential zones where it was significantly highest at *P < 0.05* (Fig. 2[a -f]). This represented a 400% increase in maize yield compared to the control and 108% maize yield increase over conventional fertilizers. Maize yield responses were also positive for multi-nutrient and conventional fertilizers in the low AEZs but higher for the latter. There was a positive relationship between N rates and maize yield where higher rates of N application recorded significant maize yield responses (Fig. 2[a - f]).

At 3.7 t/ha, Irish potatoes showed significant response (*P < 0.05*) to conventional fertilizers in the high potential AEZ. The responses to multi-nutrient fertilizers in this case may have been masked by the fact that farmers tend to replace conventional fertilizers with multi-nutrient fertilizers on a bag-to-bag basis, while it was evident that the latter had lower basal N content per bag. While all nutrients are important, the greatest proportion of crop yield is attributed to N and P [51]. Hence, the need to adjust application rates accordingly. These results were not conclusive enough to show fertilizer effects on bean yields as the response of this crop to fertilizer was not clear apart from the low agro-ecological zone. Hence, more data is needed to validate bean yield responses.

### 3.3 Profitability of multi-nutrient and conventional fertilizers under smallholder conditions

**Fig. 3:**
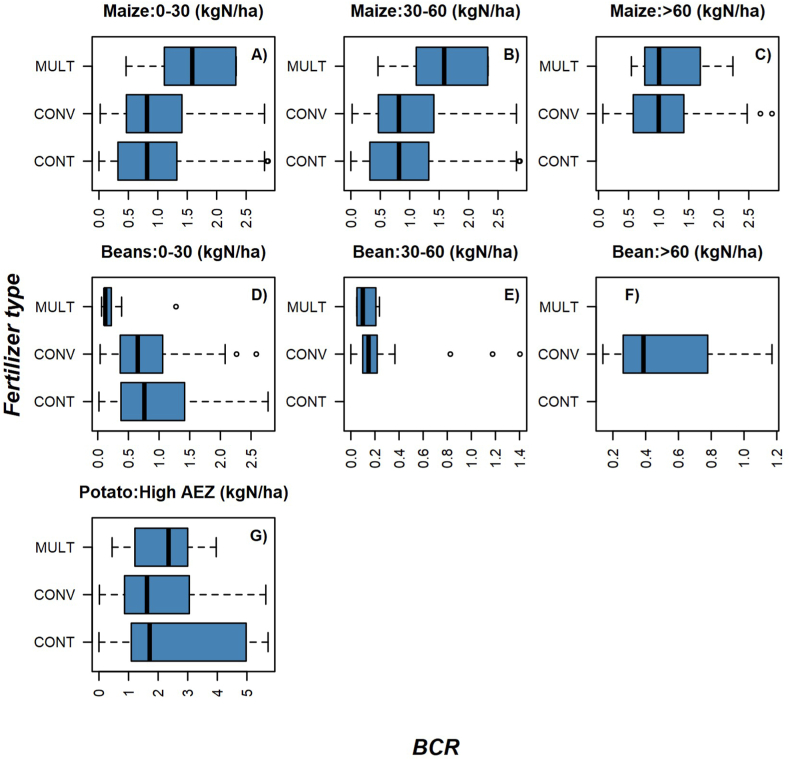
[A – G] BCR distribution for maize [0–30 kg N ha^−1^ (A), 30–60 kg N ha^−1^(B) and >60 knN ha^−1^ (C)], beans [0–30 kg N ha^−1^ (D), 30–60 kg N ha^−1^(E) and >60 kgN ha^−1^ (F)] and Irish potato (G) for different fertilizer types. Fertilizer types are truncated in upper case due to space considerations as follows; CONT-Control, CONV=Conventional, MULT = Multi-nutrient.

**Fig. 4:**
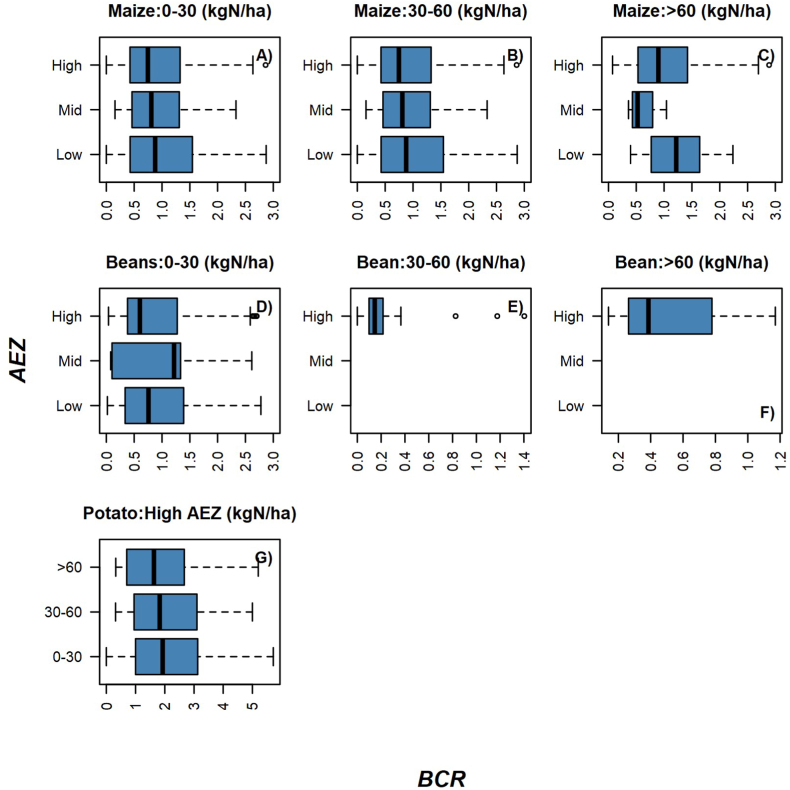
[A – G] BCR distribution for maize [0–30 kg N ha^−1^ (A), 30–60 kg N ha^−1^ (B) and >60 kgN ha^−1^ (C)], beans [0–30 kg N ha^−1^ (D), 30–60 kg N ha^−1^(E) and >60 kgN ha^−1^ (F)] and potato (G) in different agroecological zones.

**Fig. 5:**
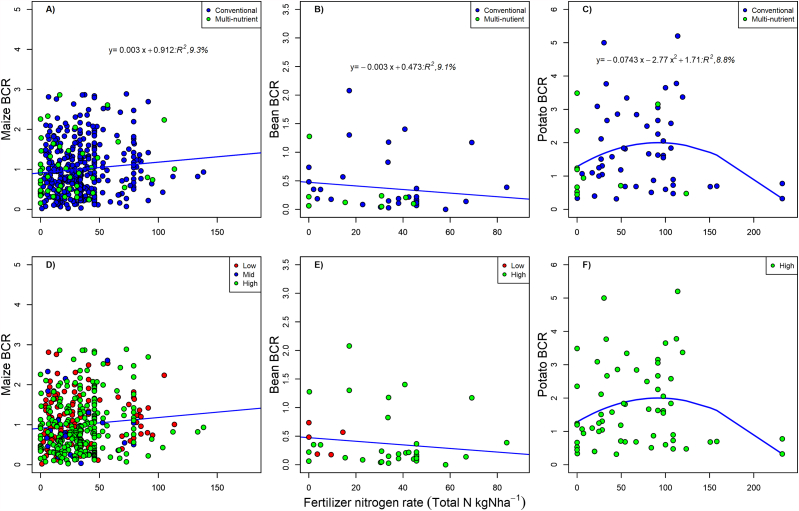
[A – F] Regression for fertilizer nitrogen rate and BCR for conventional and multi-nutrient fertilizers [maize, Figure A; beans, Figure B; Potato, Figure C] and AEZ [maize, Figure D; bean, Figure E; Potato, Figure F]. The line is the average regression line for all categories.

The benefit-cost ratio analysis showed that farmers who used multi-nutrient fertilizers on maize may break even, but their enterprise cannot be considered profitable (Fig. 3[A – G]). Given the current low use of multi-nutrient fertilizers (Table 4), their profitability on maize may become clearer as more farmers begin to use them. Conversely, the analysis showed that for potato production, multi-nutrient fertilizer use at a rate of 0–30 kg N ha^−1^ is profitable with a benefit: cost ratio (BCR) of above 2 (Fig. 3[A – G], Fig. 4[A – G]). A polynomial response in the profitability of potato cropping was observed with diminishing returns at higher levels of fertilizer N rates (>100 kgN ha^−1^) (Fig. 5 [A – F]). Poor response of fertilizer use on profitability was observed with bean crops although the farmers in the mid agro-ecological zone could break even (Fig. 4[A – G]). Recent increases in global and local fertilizer prices may have affected fertilizer use and profitability, particularly for legume crops.

### 3.4 Drivers of multi-nutrient fertilizer adoption

Factors influencing adoption of multi-nutrient fertilizers are summarized in Table 7. Financial resource allocation to farm inputs was a major determinant to the use of multi-nutrient fertilizers as seen in the farmer's monthly expenditures. A higher spending ability increases the likelihood of farmer adoption of multi-nutrient fertilizers. The location of the household is important for the uptake of these fertilizers. Households located in the high potential agro-ecological zones were more likely to use multi-nutrient fertilizers compared to those in the low potential ones. Historically, counties in high potential zones have always been better endowed than others in terms of infrastructure implying increased access to better input/output prices, good roads, and communication facilities. Households that were food secure and had access to nutrition information were likely to use multi-nutrient fertilizers. This points to a correlation between farmer uptake of multi-nutrient fertilizers and food security and nutrition security, and its awareness. Male-headed households with fewer adult members were also more likely to use these fertilizers than female-headed ones. Owning fewer livestock increases the likelihood of multi-nutrient fertilizer use. From the point of view of intensification this would make sense. Farmers with less access to farmyard manure due to few or no livestock may have opted to intensify by adopting multi-nutrient fertilizers. Institutional factors such as access to credit were also crucial in driving the uptake of multi-nutrient fertilizers.

**Table 7:** Probit model estimates of factors influencing the uptake of multi-nutrient fertilizers in Kenya.

	Coefficient	Robust standard error
Soil fertility (1 = Highly fertile)	0.01	0.14
Slope (1 = Steep)	−0.07	0.16
Adult HH members (no.)	−0.11	0.04***
Gender of HH head (1 = Female)	−0.22	0.10**
Age of HH head (years)	1.56e^−03^	2.97e^−03^
Years of education	2.46e^−03^	9.66e^−03^
Monthly expenditure (Kshs)	1.34e^−05^	3.21e^−06^***
Parcel size (acres)	−0.03	0.02
TLU	−0.04	0.01***
Electricity access (1 = HH has access)	0.06	0.08
Association membership (1 = HH in an association)	−0.04	0.09
Training (1 = HH has received agricultural training)	−0.05	0.09
Credit (1 = HH has obtained credit)	0.24	0.10**
County	0.19	0.03***
Agroecological zone (1 = High, 2 = Mid, 3 = Low)	−0.38	0.07***
Food security (1 = HH is food secure)	0.41	0.08***
Nutrition (1 = HH has accessed nutrition information)	0.17	0.09*
_constant	−0.15	0.33
Observations	2156	
Wald chi 2 (14)	562.80	
Pseudo R2	0.31	

***Significance at 1% level, **at 5% level, and *at 10% level.

## 4 Discussions

As SSA's population continues to expand rapidly, it is imperative that the continent devices ways of feeding its populace without depending on food aid, imports or depleting valuable foreign exchange reserves. In 2018, Kenya imported 0.57 million tonnes of maize (and maize products), 0.14 million tonnes of sorghum (and sorghum products), and 1.82 million tonnes of wheat (and wheat products; http://www.fao.org/faostat/en/#data/FBS). Overall, the country spent about 2.3 billion US dollars on food imports, with cereals accounting for 41% of this amount [52].

From the preceding analysis, multi-nutrient fertilizers have the potential to increase on-farm crop yields. While the use of multi-nutrient fertilizers on maize boosted productivity in the high potential areas, conventional fertilizers improved potato productivity in those zones. Given the predominant role of maize as the country's staple crop, the use of multi-nutrient fertilizers is a viable means through which food and nutritional security for all households could be achieved. Use of multi-nutrient fertilizers has the potential of alleviating “hidden hunger” caused by deficiency of some minerals such as zinc, iron, copper, and manganese in the foods consumed. Globally, the problem of micronutrient deficiencies or ‘hidden hunger’ is even more perverse than hunger. The prevalence of chronic malnutrition among young children in Kenya is estimated at 30% [53,54]. Diseases associated with malnutrition include stunted mental and physical growth, anaemia, and impaired immunity and night blindness [[18], [19], [20]]. Food crops in Africa do not contain adequate micronutrients (e.g., grain Zn) for adequate human nutrition [22] due to soil and geological deficiencies. Agronomic biofortification through micronutrient fertilizer application to food crops has the capacity to not only raise crop productivity but also to improve nutritional quality and reduce micronutrient deficiencies [16,19,22]. The recommended daily intake of zinc, a major limiting micronutrient in human diets, is 15 mg [55]. This implies that closing yield gaps with the appropriate fertilizers, in this case multi-nutrient fertilizers containing Zn, to a large extent reduces malnutrition.

However, the likely contribution of agronomically applied micronutrients to human nutritional status requires much more information such as the micronutrient retention after storage, bioconversion, and bioavailability of ingested nutrients, processing, and cooking techniques among others [56]. Given the prevailing situation where only a small fraction of farmers in the study counties use multi-nutrient fertilizers (Table 4), there is still much to be done to raise awareness on the benefits of using such fertilizers if hidden hunger is to be addressed in Kenya and the SSA region. Even so, the formulations of multi-nutrient fertilizers should contain adequate macronutrients (e.g., N, P and K), which are often yield limiting, for optimum results [51]. Appropriate rates of application per unit area of various plant nutrients should therefore be well displayed on the packaging of multi-nutrient fertilizers to enhance application of appropriate fertilizer quantities.

The economic viability of multi-nutrient fertilizers is important for their widespread use. Our analysis shows that multi-nutrient fertilizers increased on-farm profitability of crops. While farmers may break even when they use of multi-nutrient fertilizers on maize particularly in the low potential areas, their profitability is more apparent in potato production systems which are important income earners for farmers in high potential areas. Technologies generated from agronomic trials are considered economically viable when they have at least a 100% rate of return i.e., a 2 to 1 benefit: cost ratio [5]. A benefit: cost ratio of 2 is preferred when examining fertilizer profitability as this accounts for risk concerns among farmers and any unobserved costs attached to its use [57]. Benefit: cost ratio is a function of input costs, crop yields and their market prices. Hence, fluctuations in produce market prices and yields determine whether the BCR will meet the threshold of 2. This was corroborated by Ref. [1] in their study on profitability of high input-low input farming systems in the central highlands of Kenya. High BCRs (up to 2.2) were attained with a sharp rise in prices. Nevertheless, biophysical factors such as erratic rainfall and inherent soil infertility and others such as farmer preferences and attitudes, management skills and labor dynamics may preclude this even when prices are high [1,57].

Given the low use of multi-nutrient fertilizers countrywide, their on-farm profitability will become clearer with increased intensification, improved uptake of appropriate recommendations and adoption of the 4 R framework for sustainable fertilizer application, i.e., using the right sources and rates of fertilizer that are applied at the right time and place [15]. Therefore, there is scope for these fertilizers to be also profitable in maize systems with higher uptake and upward price movements of the staple crop on the market. There has been a recent steep increase in maize output prices on the Kenyan market (https://www.businessdailyafrica.com/bd/data-hub/farmers-reap-from-historic-maize-price-as-crisis-looms--4022366). This is certainly balanced out with the current spike in input prices [58,59], but if fertilizers are applied at reasonable rates (0–30 kg N/ha or 30–60 kg N/ha) it may still be possible to realize some profit. The relatively low share of households that can access credit points to a failure of institutions to play a supporting but crucial role in driving agricultural development in Kenya. Thus, socio-economic, and institutional considerations are important when assessing whether multi-nutrient fertilizers can be used at scale. The location of a household matters from a policy perspective given that policy incentives vary across counties. The county government of Kakamega, for instance, has been supplying subsidized multi-nutrient fertilizers to its farmers since 2017 (https://kakamega.go.ke/county-government-subsidises-farming/). This in effect has not only increased exposure to the fertilizers but also led to their widespread use in the county relative to other counties apart from Bungoma.

## 5 Conclusions

Amidst the prevailing global food crisis and skyrocketing fertilizer input costs, this study furnishes a unique micro-perspective on the use, response, and profitability of multi-nutrient fertilizers in Kenyan smallholder farms. Our results show that multi-nutrient fertilizers increase crop yields and profitability on Kenyan smallholder farms relative to conventional fertilizers. However, yield response as well as the economic viability of multi-nutrient fertilizer use varies with agro-ecological zones and the crop under production. Multi-nutrient fertilizers are important for improving maize yields in the high potential zones hence considerably improving the country's prospects of becoming food-secure in terms of its major staple crop. Also, multi-nutrient fertilizers when used in potato production systems are profitable and at least smallholder farmers get to break even when they use these fertilizers on maize production systems. The results showed positive bean yield response to application of fertilizers at higher rates in the low potential agro-ecological zones. However, more research needs to be conducted in legume (common bean) cropping systems as results are not conclusive enough to show fertilizer effects on bean yields as well as the profitability of fertilizer application on this crop. Still, our results point to good bean response to fertilizer at higher rates in the low potential zone. Therefore, more research on bean response to fertilizer application and corresponding yields and BCR need to be directed to the low potential Tharaka Nithi, Makueni and Kitui counties.

Currently, the uptake of multi-nutrient fertilizers is low across the country. Hence, there should be concerted efforts to roll out multi-nutrient fertilizers accompanied by extension messages about appropriate rates of application of various soil nutrients including the secondary nutrients (S, Mg, Ca) and micro-nutrients (Zn, B, Mg, Se). Our analysis shows there is considerable potential for multi-nutrient fertilizers to increase crop productivity while being economically viable across AEZs and cropping systems. Going forward, similar assessments undergirded by farmer-centric experimentation need to be encouraged for in-depth understanding of the underlying factors behind adoption of multi-nutrient and conventional fertilizers and their contributions towards food and nutrition security in Kenya.

## Author contribution statement

Ivan Solomon Adolwa: Conceived and designed the experiments; Analyzed and interpreted the data; Wrote the paper.

James Mutegi: Conceived and designed the experiments; Wrote the paper.

Joses Muthamia; Angela Gitonga: Performed the experiments; Wrote the paper.

Samuel Njoroge: Contributed reagents, materials, analysis tools or data; Wrote the paper.

Abednego Kiwia; Eileen Bogweh Nchanji : Contributed reagents, materials, analysis tools or data; Dismas Manoti: Performed the experiments.

Franklin Mairura: Analyzed and interpreted the data; Contributed reagents, materials, analysis tools or data.

## Data availability statement

Data will be made available on request.

## Declaration of competing interest

The authors declare that they have no known competing financial interests or personal relationships that could have appeared to influence the work reported in this paper.
