# Supplementary material for: Enhancing sustainable agri-food systems using multi-nutrient fertilizers in Kenyan smallholder farming systems
Source: Heliyon. 2023 Apr 11;9(4):e15320. doi: 10.1016/j.heliyon.2023.e15320 (PMC10161611; doi:10.1016/j.heliyon.2023.e15320)
Supplement: Multimedia component 1 [file mmc1.pdf]

Agriculture and Food Security Study 2020

Tool for Data Collection from Small-holder Farmers in Kenya

This tool will be administered to ***smallholder farmers*** *(farmers with landholdings of not more than 10 acres or those who have rented land of not more than 10 acres ...)*

Section A: Introduction (interviewer/translator) and identifying variables

"Good morning/afternoon. We are part of an APNI research team, conducting an agriculture and food security analysis in several counties in Kenya. We would like to understand how you use fertilizers, improved seeds, other agricultural technologies, and how they are relevant to your nutrition and wellbeing. For this purpose, we will ask you a few questions and guide the discussion. The information you provide will be used purely for research purposes. Your name will not appear in any data that is made publicly available. Do you consent to be part of this study? If there are questions that you would prefer not to answer, then we respect your right not to answer them."

Household ID ………………………

A1. Has consent been given? Yes *(1)* No (0)
A2. Date of interview (day/month/year) _ _/_ _/_ _ _ _
A3. Start time (hrs: mins)

A4. County of study: (1) Makueni (2) Kitui (3) Tharaka-Nithi (4) Meru (5) Siaya(6) Uasin Ngishu (7) Meru (8) Kakamega 9) Bungoma

A5. SubCounty of study: (11) Makueni subcounties –Makueni, Mbooni, (21) Kitui sub counties (Mwingi Central, Kitui West, Kitui Rural) (31) Tharaka Nithi subcounties –Maara, Chuka Igamba ngómbe, Tharaka North, (41)Meru subcounties-Tigania West (maize); Buuri East (Potatoes); Buuri West (Potato); Central Imenti (Maize)…. (51)Siaya subcounties: Gem, Ugunja….(61) Uasin Gishu- Ainabkoi, Kesses, ….(71)….(81) Kakamega –Lurambi, Mumias east,….(91) Bungoma –Bumula, Kabuchai…..(101)….(111)….

A6. Ward of study: (11) Makueni subcountiesward: Makueni (Wote, Muvau/Kikumini), Mbooni(Kako/Waia, Mbooni) (21) Kitui sub countiesWards -Mwingi Central (Kivou, Nguni), Kitui West (Mutonguni, Kithumula), Kitui Rural (Kwa Vonza) (31)Tharaka Nithi subcounties –Maara- Ganga, Mwibi; Chuka Igamba ngómbe- Magumoni, Mariani, Tharaka North-Mukothima, (41)Meru subccountiesWards-Tigania West-Nkomo; Buuri East-Kiirua, Kibirichia; Buuri West-Timau; Central Imenti –Abo central;

(51)Siaya subcounties: Gem-(North Gem,Central Gem), Ugunja (Sigomere, Ugunja)…. ….(61) Uasin Gishu subcoutywards-Ainabkoi (Kaptagat, Olare), Kesses (Cheptiret, Tarakwa),)….(81) Kakamega Subcountieswards –Lurambi (East Butsotso, Central Butsotso), Mumias east (Lushea/Lubino, East Wanga),….(91) Bungoma subcountieswards–Bumula (Siboti, South Bukusu), Kabuchai (Malaka, Nalondo West)

Village of study (make the village a string) (111)
(211) (212) (213)

A7. Interviewer (enumerator) name: 1= 2= 3=; 4=;

A8. Supervisor name: 1= 2= 3=

Section B: Questions on the household composition.

How many people live together with you in this household (HH; i.e. those sharing the same house and regularly taking meals in the last 12 months)? List all the adult household members i.e., 18 years and above. For children (those under 18), their number should be aggregated with the rest in **B6**.

Fill the form below:

**B1.** Name of HH member (start with respondent)	**B2.** Are you a decision maker of your household? (*Yes=1, No=0*)	**B3.** Relation to HH head **Use code B3**	**B4.** Gender of HH members (1=female;0=male)	**B5.** Age (years) **Use code B5**	**B6.** How many people live in your household?	**B7.** No. of years of formal education **Use code B7** *(for respondent/HH only)*	**B8.** Years of farming experience *(for respondent/HH only)*	**B9.** Average monthly expenditure (Kshs)	**B9.** Main occupation **Use code B9**	**B10.** Monthly net income in Kshs if NOT farming	**B11.** 2nd important occupation **Use code B9**	**B12.** Monthly net income in Kshs if **NOT** farming	**B13.** Other income sources **Use code B13**	**B14.** Monthly net income from other sources (Kshs)

Code B3:
1 Household head
2 Spouse
3 Son/daughter
4 Parent
5 Son/daughter in-law
6 Grand child
7 Other relative
8 Other, specify……

Code B5:
1 <18
2 18-24
3 25-34
4 35-44
5 45-59
6 60-74
7 >74

Code B7:
0 None (illiterate)
1 Basic (can write and read)
2 Lower primary (1-4)
3 Upper primary (5-8)
4 Junior secondary (9-10)
5 Senior secondary (11-12)
6 Vocational training
7 College
8 University
9 Other, specify …

Code B9:
0 No occupation
1 Farming (crop and/or livestock)
2 Herds boy/girl
3 Housekeeping
4 Casual labourer on another farm
5 Non-farm business (shops, trade, tailor, etc)
7 Salaried employment
8 Other, specify………
9 Student

Code B13:
1 Rented out land
2 Rental income (e.g. from renting tractor, animals for traction, houses)
3 Sale of dung cake for fuel
4 Sale of own trees (firewood, etc)
5 Own business
6 Pension income
7 Drought relief
8 Remittances (sent from non-resident family and relatives)
9 Marriage gifts (e.g., dowry)
10 Other, specify ……………..

Section C: Number of cropped plots and plot characteristics

C1. What is the total number of fields cropped in the last Long rain and Short rain cropping season? (Enumerator: Be careful that the farmer does not treat entire farm as a single field)

**C11.** Field numb er	**C12.** Field location name (as called by farmer:s tring)	**C13.** Field distance from residence (Walking min.)	**C14.** Plot size (Acres)	**C15.** Slope of the plot 1=Gentle slope (flat) 2=Medium slope 3=Steep slope	**C16.** Soil fertility 1=Poor 2=Medium 3=high	**C17.** Soil type (String variable: Insert soil type including local descriptions)	**C17.** Crops grown in [plots] (See code sheet) **Multi-choice**	**C18.** Specify type of ownership **Use code C18**

Code C18: 1=Owned with Land title, 2= Owne without land title , 3=Leased out, 4= Rented, 5= Share-cropped, 6=Other

Section D: Crop production and management

i. *Tell us about your crop production and management practices in the previous long-rain (LR) and short-rain (SR) seasons.*

**D1.** Field number	**D2.** Crop Code (See crops code sheet on last page)	**D3.** Area under this crop (acres)	**D4.** Season (LR or SR)	**D5.** Date of sowing		**D6.** Spacing 0=ad-hoc 1=recommended	**D7.** Spatial arrangement **Use Code D7**	**D8.** If there is spatial arrangement, with which crop? (See crops code sheet)	**D9.** Weeding 0=none 1=once 2=twice 3=thrice 4=other	**D10.** Harvest		**D11.** Sales		**D12.** Revenue	
				Month/Year	Week					Qty	Unit **Use code D10**	Qty	Unit **Use code D10**	Average unit Price eg 90 kg bag (Kshs)	Gross sales (Kshs)

Code D7
0=Mono cropping
1=Intercropping
2=Crop rotation
3=Others (Specify…)

Code D10:
1=90 kg bag
2=50 kg bag
3=Kgs
4=Litre
5=Gorogoro
6=Tonnes
7=Debe
8=Crate
9=Numbers/pieces
10=Bunch(bananas)
11=Grams
12=25 kg bag
13=10 kg bag
14=2kg bag
15=Other (Specify…)

ii. *Which crop production inputs did you use in the previous long-rain and short-rain seasons?*

<table>
<tr><td colspan="5">D13. Seed use</td><td colspan="8">D14. Mineral Fertilizer Use</td><td colspan="5" rowspan="3">D15. Organic Fertilizer Use</td><td colspan="4" rowspan="2">D16. Applied lime
1=Yes, 2=No</td><td rowspan="3">D17. Expenditure on pesticides (herbicides/ insecticides/ fungicides)
Kshs</td></tr>
<tr><td rowspan="3">Seed type?
1=Local
2=Improved
3=Mixed
4=Saved seeds</td><td rowspan="3">If improved, what variety?
Name: String</td><td rowspan="3">Qty</td><td rowspan="3">Unit
Use code D10)</td><td rowspan="3">Price per unit Kshs</td><td colspan="4" rowspan="2">D141. 1st Mineral fertilizer application</td><td colspan="4" rowspan="2">D142. 2nd Mineral fertilizer application</td></tr>
<tr><td colspan="4">D161. If Yes,</td></tr>
<tr><td>Type
Use code D14</td><td>Qty</td><td>Unit
Use code D10</td><td>Unit price Kshs</td><td>Type
Use code D14</td><td>Qty</td><td>Unit
Use code D10</td><td>Unit price Kshs</td><td>Type
Use code D151</td><td>If type=3, type of plant used?
Use code D152</td><td>Qty</td><td>Unit
Use code D10</td><td>Price per unit Kshs</td><td>Type
Use code D16</td><td>Qty</td><td>Unit
Use code D10</td><td>Unit price Kshs</td><td></td></tr>
<tr><td></td><td></td><td></td><td></td><td></td><td></td><td></td><td></td><td></td><td></td><td></td><td></td><td></td><td></td><td></td><td></td><td></td><td></td><td></td><td></td><td></td><td></td><td></td></tr>
<tr><td></td><td></td><td></td><td></td><td></td><td></td><td></td><td></td><td></td><td></td><td></td><td></td><td></td><td></td><td></td><td></td><td></td><td></td><td></td><td></td><td></td><td></td><td></td></tr>
</table>

Code D10:
1=90 kg bag
2=50 kg bag
3=Kgs
4=Litre
5=Gorogoro
6=Tonnes
7=Debe
8=Crate
9=Numbers/pieces
10=Bunch(bananas)
11=Grams
12=25 kg bag
13=10 kg bag
14=2kg bag
15=Other (Specify…)

Code D14:
1=NPK (23:23:0)
2=NPK (17:17:17)
3=Baraka standard Planting
4=Baraka standard topdess
5=Baraka legume
6=Baraka Standard topdress
7=Baraka Potato planting
8=Baraka Potato Topdress
9=Baraka Legume planting
10=Baraka Horticulture
11= Fanisi Planting
12=Fanisi Topdressing
13=Kyno Nafaka
14= KynoPlus S
15=Kynoplus Top
15=DAP
16=CAN
17=MEA NPK 10:26:10
18=NPS (OCP)
19=NPSB (OCP)
20=UREA (46:0:0)
21=Folia Feeds
22=Mavuno Planting
23=Mavuno-Top Dress
24=Mijingu Nafaka
25=YaraMila Winner
26=YaraMila Power
27=Yara Mila Cereals
28=YaraVera Amidas
29= YaraBela Sulfan
30=YaraMila Cereal
31=YaraBela Extran
32=Other specify (___)

Code D151:
0= None
1=Farmyard manure
2=Animal Manure
3=Green Manure
4=Compost
5=Biochar
6=Other specify (__________)

Code D152:
1=Tithonia
2=Calliandra
3=Mucuna
4=Desmodium
3=Other specify (_________)

Code D16:
1=Agricultural lime
2= Dolmax
3= Calciprill
4= Magprill
5= Dolomitic lime
6=Calcitic lime
7=Do not know
8=Other (Specify…)

iii. *What other costs did you incur in the past long and short rains for your farm production? Please estimate your expenditures in Kshs*

**D17.** Transportation costs	**D18.** Sacks	**D19.** Labor (farm workers; total wages/cash expenditures)	**D20.** Hired equipment/tractor	**D21.** Hired oxen	**D22.**Cost of rented land	**D.23** Other (specify)

Section E: Livestock ownership and production

If you own livestock, please list the no. of animals that you own, and the production and utilization of livestock products (in the last short and long-rain seasons)

**E1.** Ownership			**E2.** Production and consumption						
**E11.** Animal type<br>**Use code E11**	**E12.** Current Stock 2020 (No.)	**E13.** Estimated Value (Kshs)	**E21.** Livestock product<br>**Use code E21**	**E22.** Frequency of production<br>**Use code E22**	**E23.** Quantity produced	**E24.** Units of production<br>**Use code E24**	**D25.** Quantity consumed	**D26**. Quantity sold	**D27.** Total revenue (Kshs)

Code E11:
1=Cow
2= Heifer
3= Bull/Oxen
4= Calf
5= Goat
6= Sheep
7= Pig
8= Chicken/Poultry
9=Donkey
10=Horse
11= Fish
12= Bee (hive)
14= Camel

Code E21:
1= Milk
2=Eggs
3=Animal skin
4=Honey
5=Meat
6=Animal manure
7=Other, specify…

Code E22:
1=Daily;
2=Weekly;
3=Monthly;
4=Every 3 months;
5=Every 4 months;
6=Every 6 months;
7=Annually;
8=Other, specify……..

Code E24:
1=Litres;
2=Kg;
3=Pieces;
4=Trays;
5=Other, specify……….

Section F: Housing characteristics and assets

**F1.** Asset type **Use code F1**	**F2.** Number owned	**F3**. Do you have electricity access at home? 1=Yes; 2=No	**F4.** Does your home have glass windows? 1=Yes; 2=No	**F5.** What is the main **walling** material of main residential house? **Use code F5**	F6. Source of drinking water **Use code F6**	**F7. Distance** to main source of water in walking time? (minutes)	**F8.** What kind of toilet facilities does your household use? **Use code F8**	**F9.** What is the main **roofing** material for the main residential house? **Use code F9**

Code F1:
1=Tractor
2=Trailer
3=Vehicle
4=Motorcycle
5=Wheelbarrows
6=Ploughs
7=Borehole
8=Knapsack sprayer
9=Solar panels
10=Bicycle
11=Radio
12=Television
13=Mobile phone
14=Water tanks
15=Beehives
16=Motorized pump
17=Other (specify)

Code F5:
1=Burned bricks
2=Mud bricks
3=Stone
4=Planks
5=Timber with mud
99=Other (specify)

Code F6:
1=Piped inside the house
2=Piped outside the house
3=Borehole protected and covered
4=Well

Code F8:
1=Water cistern
2=Pit latrine
3=Shared pit latrine
4=Communal pit latrine
5=No toilet/bush/field

Code F9:
1=Iron sheet
2=Grass
3=Both, iron sheet and tiles
4.=Brick tiles
99=Other (specify)

Section G: Questions on the knowledge and adoption of varieties, fertilizers (conventional and blended), and management practices for the past LR and SR seasons (limit to maize, green grams, potatoes, and common beans)

i) *Improved crop varieties*

**G1.** Seed varieties, known **Name: String**	**G2.** Type of variety, (**0**=Local; **1**=Improved)	**G3.** For all **improved** varieties known				**G4.** Year first planted	**G5.** Reasons for planting **Use code G3311**	**G6.** Main source of first seed? **Use code G6**	**G7.** Where did you see the variety grow in the field before adopting? **Use code G7**	**G8.** Planted or used variety in? 2019SR (0=N0; 1=Yes)	**G81.** If No, why not **Use code G81**, rank 3)	**G9.** Planted or used variety in 2020LR (0=N0; 1=Yes)	**G91.** If No, why not **Use code G81**, rank 3)
		**G31.** When (year) did you first hear about the variety?	**G32.** From whom/where did you first hear about it? Rank up to three **Use code G32**	**G33.** Ever planted the variety? **(0=no; 1=yes)**	**G331.** If **YES,** give reasons **Use code G3311**								

ii) *Fertilizers (conventional and blended)*

**G10.** Fertilizers, known **Use code D14**	**G11.** Type of fertilizer (**0**=conventional; **1**=blended)	**G12.** For all **blended** fertilizers known				**G13.** Year first used	**G14.** Reasons for using **Use code G331**	**G15.** Main source of first fertilizer **Use code G6**	**G16.** Used fertilizer in 2019SR (0=N0; 1=Yes)	**G17.** If No, why not **Use code G81**, rank 3)	**G18.**Used fertilizer in 2020LR (0=N0; 1=Yes)	**G19.** If No, why not **Use code G81**, rank 3)
		**G121.** When (year) did you first hear about the fertilizer?	**G122.** From whom/where did you first hear about it? Rank up to three **Use code G32**	**G123.** Ever used the fertilizer? **(0=no; 1=yes)**	**G1231.** If **YES,** give reasons **Use code G331**							

iii) *Management practices*

**G20.** Management practices known **Use code G20**	**G21.** For all **management practices** known				**G22.** Year first used	**G23.** Reasons for using **Use code G331**	**G24.** Main source of practice **Use code G6**	**G25.** Where did you see the being used in the field before adopting? **Use code G7**	**G26.** Used practice in 2019SR (0=N0; 1=Yes)	**G27.** If No, why not **Use code G81**, rank 3)	**G28.** Used practice in 2020LR (0=N0; 1=Yes)	**G29.** If No, why not **Use code G81**, rank 3)
	**G211.** When (year) did you first hear about the practice?	**G212.** From whom/where did you first hear about it? Rank up to three **Use code G32**	**G213.** Ever used the practice? **(0=no; 1=yes)**	**G2131.** If **YES,** give reasons **Use code G331**								

Code G20:
1=Lime application
2=Heaping or ridging
3=Crop residue use
4=Manure application
5=Minimum tillage
6=Mulching
7=Intercropping
8=Crop rotation
9=Other, specify

Code G32:
1=Government extension
2=Farmer-based organisation
3=NGO
4=Research centre
5=On-farm trials/demos/field days
6=Seed/grain stockist
7=Another farmer/neighbor
8=Radio/newspaper/TV
9=Farmer magazine
10=Mobile phone updates
11=Drama/skit
12=Other, specify…..…

Code G331:
1=No other variety/fertilizer/management available
2=Best adapted variety
3=High yields
4=……. … (*Please fill name*) recommended it to me
6=Drought tolerance
7=Early maturity
8=Sweet taste/aroma
9=Good flour quality
10=Brewing quality
11=High price
12=Other, specify

Code G6:
1=Research
2=Extension officer
3=Bought from local seed producers
4=Bought from local trader or agro-dealers
5=Farmer to farmer seed exchange (relative, friend, etc)
6=Provided by NGOs
7=Other (specify)…

Code G7:
0=Never seen it grow before adopting
1=Within my villager
2= Neighboring village
3= Research station
4=. Demo/trial plot
5= Agricultural show
6= Other (specify)…

Code G81:
1=Cannot get seed at all
2=Lack of cash to buy seed/fertilizer
3=Susceptible to field pests/diseases
4=Susceptible to bird attack
5=Susceptible to storage pests
6=Poor taste
7=Cannot get credit
8=Low yielding variety
9=Labor-intensive
10=Too complex
11=Other, specify….

Section H: Social capital and networking

**H1.** For how many **years** have you been **living** in this village? *(If the respondent is Head of the HH, go to H2)*	**H2.** Number of **years** the Head of the HH has been **living** in this village?	**H3.** Name a maximum of 3 persons with whom you frequently discuss agricultural matters	**H4.** How is he/she related to you? **Use code H4**	**H5.** Which institution are they affiliated to? **Use code H5**	**H6.** Do you or any of your family members belong to an association ? (fill in all that apply, **code H6**)	**H7.** What specific information regarding improved varieties or fertilizer blends have you : **Use code H7**		**H8.** Who else do you discuss agricultural issues with? (Name)	**H9.** Which institution are they affiliated to? **Use code H5**	**H10.** How often do you discuss agricultural matters with this person? **Use code H10**
						Given to this person	Received from this person			
		1.								
		2.								
		3.								

Code H4:
1=Parent;
2=Child;
3=Brother/sister; 4=Grandparent;
5=Grandchild; 6=Nephew/Niece;
7=Uncle/aunt;
8=Cousin;
9=Same family lineage;
10=Mother/father in-law;
11=Brother/sister in-law;
12=Other relative;
13=Fellow villager/Neighbor;
14=Attend same church/ mosque;
15=Professional/business colleague;
16=Other, specify…….

Code H5:
1=Government extension (MoFA)
2=Farmer-based organisation
3=NGO
4=Research centre
5=Government department
6=Seed/grain stockist
7=Another farmer/neighbor
8=County government
9=Marketer
10=Media
11=Credit organisation
12=Other, specify…...…

Code H6:
0=No;
1=Farming group;
2=Self-help group;
3=Merry go round;
4=Savings and Credit;
5=Labour cooperative;
6=Other (Specify)………

Code H7:
1=Information on hybrid maize seed varieties
2=Information on hybrid bean seed varieties
3=Information on hybrid potato seed varieties
4=Information on hybrid common bean varieties
5= Information on hybrid greengram varieties
6=Information on multi-nutrient fertilizer types
7=Information on fertilizer rates
8=Information on multi-nutrient fertilizer prices
9=Information on multi-nutrient fertilizer sources
10=Information on fertilizer application
11=Information on fertilizer time of application
12= Other, specify……

Code H10:
1=Occasionally
2=Moderately frequently
3=Frequently

Section I: Access to institutional services, credit, and agricultural inputs

**I1.** In the past 12 months, did you or any of your HH members receive training on fertilizer or seed use<br>1= Yes,<br><br>0=No >> go to **I2**	**I11.** If yes, list the areas/subjects for which training was received?<br><br>**Use code I2**	**I12.** How many times was training received?<br><br>(***consider total for the HH members***)	**I13.** Who in the household attended?<br><br>**Use code B3 in part B.**<br><br>*Multiple choice*	**I14**. What was the source of training<br><br>**Use code I14**	**I2.** In these **past 12 months**, did you obtain any **agricultural credit** for your crop production (all crops)?<br><br>1=YES> **I3**<br><br>2=NO	**121.** If you did not obtain credit, please tell me the main two reasons for this<br><br>**Use code I21**		**I3.** For what purpose did you use this credit? (list up to three)<br><br>1=Buy fertilizer<br>2=Buy pesticide<br>3=Buy seeds<br>4=Buy farm equipment<br>5=Land preparation<br>6=Weeding<br>7=harvesting<br>99=Other purpose (specify)			**I4.** Source of Credit<br><br>**Use code I4**
						First	Second	First	Second	Third	

Code I2:
1=New crop variety
2=Planting time;
3=Market access, value chains
4=Post-harvest handling
5= Nutritional value of crop
6=Gender integration in crop production
7=Multi-nutrient Fertilizers
8=others (specify)

Code I14:
0=I never obtained training
1=Local input stores
2=Gov't. Extension Agent
3=NGOs
4=Research Institute
99=Other (specify)

Code I21:
1=No need
2=Borrowing is too risky
3=Interest rate is too high
4=Too much paper work/procedures
5=Expected to be rejected, so I did not try it
6=I have no assets for collateral
7=No money lenders in this area for this purpose
8=Lenders don't provide the amount needed
9=No credit association available
10=No financial institution in the region
99=Other (specify)

Code I4:
1=Money lender
2=Farmer group/cooperative
3=Neighbor
4=Microfinance
5=Bank
6=Relative
7=Savings group
99=Other (specify…)

Section J: Food security and nutrition module

JI. Was the food harvested from the previous season able to last you up to the next harvest? **(1=Yes; 0=No)**

J2. Normally, in which months of the calendar year is it necessary to buy food i.e., the hunger months? (List _______string)

**J3.** During these hunger months,	**J311.** 1=Yes >> J12, 2=No >> next food security question	**J312.** How often did it occur? 1=not often, 2=moderately often, 3=often, 4=very often	**J313.** Why did this occur? **Use code J313**
**J31.** does the household head worry that they will not get enough food?			
**J32.** are you or any household member not able to eat the kinds of foods you prefer because of a lack of resources?			
**J33.** does you or any household member have to eat a limited variety of foods due to a lack of resources?			
**J34.** does you or any household member have to eat some foods that you really do not want to eat because of a lack of resources to obtain other types of food?			
**J35.** does you or any household member have to eat a smaller meal than you feel you need because there is not enough food?			
**J36.** does you or any household member have to eat fewer meals in a day because there is not enough food?			
**J37.** is there ever no food to eat of any kind in your household because they are not enough resources?			

Use code J313: 1=Sold all food from own production, 2=Consumed all food from own production/ stock, 3=No money to buy food, 4=No food at the market, 5=Other (specify…)

J38. Is there any other way you or members of your household cope with hunger? (1= Sell household assets; 2= Sell livestock/poultry; 3=Offer labor on other farms; 4=Harvest wild foods; 5= Diversify crops grown on farm; 6=Borrow from friends, relatives, neighbors; 7=Borrow from local final institutions; 8=Borrow from mobile phone lenders; 9= Borrow from saccos, banks, etc; 10=Other (Specify….)

J4. Have you or any member of your household ever received any information on nutrition of any of the crops you grow? **(1=Yes; 0=No)** *(if No skip to K5)*

J5. If yes, for what **three main crops? (Use crop code**)… *Macro-Note*

J6. Who specifically received the information …….. *(multiple selection is possible)*
(**Use code B3 in part B***)*

J7. What kind of information did you/the person receive? *(Tell me the three kinds of information you received that are most important to you)*
INFO1………………………………………………………………………………………..
INFO2…………………………………………………………………………………………
INFO3…………………………………………………………………………………………

J8. Which is the most important source of nutrition information for you (generally)? ………
(1=Newspaper; 2=radio; 3=Flour supplier; 4=TV; 5=Posters; 6=Mobile phone; 7=Community meetings; 8=Friends/ neighbours/church/group members; 9=Health centres/Clinic; 10=community health worker; 11= Social media; 888=other specify….)

Section K: Risk and vulnerability indicators module

K1. Has you or any member of your household experienced any tribal or political clashes in the past year? **(**1=Yes; 0=No)

K12. If yes, how did you or members of your household cope with these clashes? (Explain: String….)

K2. In your view, weather patterns in the past five years; 1=have remained stable, 2=have changed steadily 3= have changed suddenly, 4= Are erratic

K21. Has there been drought in your area in the last one year? (1=Yes; 0=No)

K22. What were the effects of the drought/floods? (String….)

K23. If yes, how did you or members of your household cope with these drought/floods? (Explain: String…..)

K3. Have you or members of your household suffered in any way as a result of the Covid 19 pandemic? (1=Yes; 0=No)

K31. If yes, explain? (String)

K32. What strategies have you or members of your household adopted to cope with effects of Covid-19 pandemic? (String)

K4. In your opinion, prices of food over the last one year; 1= have decreased sharply, 2=have decreased, 3= have remained stable, 4= have increased, 5=have increased sharply

K41. In your opinion, prices of agricultural inputs over the last one year; 1= have decreased sharply, 2=have decreased, 3= have remained stable, 4= have increased, 5=have increased sharply

K42. In your opinion, prices of agricultural commodities I sell at the market over the last one year; 1= have decreased sharply, 2=have decreased, 3= have remained stable, 4= have increased, 5=have increased sharply

K5. Is the household head or breadwinner of your household disabled? (1=Yes; 2=No)

K51. If Yes, type of disability (string)……………………….

K6. The household head or breadwinner is 1=widowed, 2=widowered, 3= Neither

K7. Is the household head or breadwinner of your household orphaned? (1=Yes; 2=No)

K8. Do you or any member of your household possess a health insurance card e.g. NHIF? (1=Yes; 2=No)

K81. If No, how do you normally cater for medical expenses within your household? 1=pay cash, 2= borrow from friends/relative/neighbors, 3=hold fundraisers, 4=visit local medicine man, 5=Other (Specify….)

K82. How far is the nearest clinic/dispensary from your homestead in Km?

L1. Mobile number of the farmer (respondent) ……………………………………….

L2. End time of the survey: (hrs : min) ………:………

L3. GPS coordinates of point (location):

GPS coordinates

GPS 1	Elevation (meters):
GPS 2	Latitude (decimal degrees):
GPS 3	Longitude (decimal degrees):

THANK YOU FOR YOUR TIME!

Crop Codes Sheet: arranged alphabetically

Code	Crop	Code	Crop	Code	Crop	Code	Crop
88		128		137		81	
43	Arrowroots	112	Peas	149		8	sorghum
140	Amaranthus	67		75	nappier /elephant grass	38	
86	Avocado	20		113		108	soyabeans
51		25		105	njahi (dolichos)	62	spinach
18		100		37	njugu mawe(bambara bean)	91	
10	Bananas	58		77		133	
141		127		73		145	
56		34	green grams	85		125	
7	Beans	115		57		131	
150		33	groundnuts	71	oranges	15	sugarcane
93		68		22		118	
117		102		130		60	sukuma wiki
82	cabbage	101		55		30	sunflower
139		27	Irish potatoes	99	passion fruit	64	
63		49		45		42	sweet potatoes
83	carrots	52		79		48	
24	cashew nuts	147		54		3	tamarind
28	cassava	78		66	pawpaws	132	
47		146		114		98	
104		70		96		12	tea
123		121		61		29	tobacco
26	chickpeas	32		103	pigeon peas	59	tomatoes
94		87		95		110	tree tomato
41	citrus	97	macadamia nuts	90		50	
23		142		126		53	
136		46		35		5	trees
135		4		72	pumpkin	109	
6	coffee	1	maize	120		144	
124		2		17		65	watermelon
11		69	mangoes	148		13	wheat
116	c	143		31	rice	40	
14	cotton	44		80		111	
21	cowpeas	89		119		107	
19		151		36		76	yams
92		9	millet	74		84	
134		106		39		122	
129		138		16			
